# Coenzyme Q10 Supplementation enhances testicular volume and hemodynamics, reproductive hormones, sperm quality, and seminal antioxidant capacity in goat bucks under summer hot humid conditions

**DOI:** 10.1007/s11259-022-09991-8

**Published:** 2022-09-01

**Authors:** Hossam R. El-Sherbiny, Elshymaa A. Abdelnaby, K. H. El-Shahat, Noha Y. Salem, Eman S. Ramadan, Shimaa G. Yehia, Mohamed Fathi

**Affiliations:** 1 Theriogenology Department, Faculty of Veterinary Medicine, Cairo University, Giza square, Giza, 12211 Egypt; 2 Department of Internal Medicine and Infectious Diseases, Faculty of Veterinary Medicine, Cairo University, Giza, Egypt

**Keywords:** Antioxidants, Coenzyme Q10, Goats, Heat stress, Reproductive hormones, Semen, Testicular blood flow

## Abstract

Oxidative stress (OS) is brought on by heat stress (HS), which weakens antioxidant defense and initiates OS. Since mitochondria are the primary source of reactive oxygen species (ROS), HS-mediated OS may be lessened by targeting mitochondria with particular antioxidants. The purpose of this study was to investigate the effect of oral coenzyme Q10 (CoQ10) supplementation on the reproductive performance of goat bucks under HS conditions. Ten mature bucks were randomly separated into two groups and housed in an environment with a high-temperature humidity index (THI: 88.3 to 94.8; summer season). The first group (*n* = 5) got the baseline diet while the second group (*n* = 5) received supplemental oral CoQ10 (3 mg/kg BW; CoQ10 group) daily for six weeks. Testicular blood flow parameters (TBF), testicular volume (TV) and echogenicity (TE), nitric oxide (NO), seminal alanine aminotransferase (ALT) and catalase (CAT) activities, total antioxidant capacity (TAC), malondialdehyde (MDA) content, and semen quality traits were all measured. The examinations started a week before (W-1), on the first supplementation day (W0), and weekly for eight consecutive weeks (W1-W8). There were marked *(P* < *0.05)* increases in TBF (W3-W6) and TV, and a decrease in TE (W3-W5) in the CoQ10 group compared to the CON group. Similarly, testosterone (T) and NO levels (W3-W5) in the CoQ10 group were higher *(P* < *0.05)* than those of the control group. The CoQ10 group demonstrated significant *(P* < 0.05) increases in seminal CAT (W4-W8) and TAC (W2-W6) activities and decreases in ALT (W4-W7) activity and MDA (W5-W8) concentration as compared to the control group. The CoQ10 group showed improvements *(P* < 0.05) at W3-W6 for sperm progressive motility, viability, and normal morphology and at W6-W8 for sperm concentration. In conclusion, oral CoQ10 supplementation improved testicular hemodynamics, testosterone production, semen quality, and antioxidant capacity in goat bucks during summer heat stress conditions.

## Introduction

Recently, there have been raising concerns regarding the global warming issue, which could affect livestock production and reproduction (Jamal et al. 2021). With the elevation of the planet's temperatures, animals would be exposed to environmental heat (HS) stress (Belhadj Slimen et al. 2016). Several studies have reported adverse effects of HS on male reproductive functions through reduced sperm quality and production, steroid production and libido, and potential fertility (Al-Kanaan et al. 2015). HS has an immediate adverse effect on sperm properties such as morphology, plasma membrane integrity, and fertility (Shahat et al. 2020). In addition, in hot and humid conditions, reproductive hormones such as gonadotropins (FSH and LH) and steroids (testosterone and estrogen) play important roles in controlling testicular processes such as spermatogenesis and sexual behavior. (Gündoğan 2007; Morrell 2020). In addition, testicular blood flow (TBF) decreases during the hot summer months as one of the most important determinants of testicular functions due to the supply of essential nutrients and oxygen (Samir et al. 2018; Hedia et al. 2020).

The goats are considered to be more tolerant to temperature fluctuations than other ruminants such as cows and sheep, via an increase in water conservation, respiratory and cardiac rates, and a decrease in metabolic rate (Lu 1989); however, this claimed adaptation is based mainly on the territory, breed under examination, and individual responses to HS (Lu 1989; Sharma et al. 2013; Salama et al. 2014). Baladi goats are more prone to heat stress than crossbreeds under subtropical conditions (Teama and El-Tarabany 2016; Al‐Dawood 2017; El-Tarabany et al. 2017). Temperature-humidity index (THI) has become the main tool to evaluate the HS status of the examined animal (Habeeb et al. 2018). It has been reported that ruminants reared in THI over 75 were considered under HS conditions (West 2003).

The deteriorating effect of HS on testicular functions is primarily caused by the production of free radicals [reactive oxygen species (ROS) and nitrogen species (RNS)] during mitochondrial oxidative phosphorylation processes and the loss of compensatory antioxidant defenses and subsequent oxidative stress (OS) (El-Tohamy et al. 2012). Hedia et al. (2020) reported a marked decrease in blood and semen antioxidant enzymes [superoxide dismutase (SOD), glutathione peroxidase (GPx), and catalase CAT)] and an increase in malondialdehyde (MDA, lipid peroxidation biomarker) accompanied by lower TBF during summer months in rams. Recently, mitochondria-targeted antioxidants such as coenzyme Q10 (CoQ10) have been artificially involved in improving OS-induced cell damage, with better results than cytosolic counterparts (Elokil et al. 2019; Tiwari et al. 2021).

CoQ10 is a fat-soluble, vitamin-like, mitochondrial origin, and bio-energetic molecule, structured with the quinone group and existing ubiquitously in all cell types in both animals and plants; this is why it is called ubiquinone. CoQ10 exists heavily in the mitochondria and is implemented in the electron transport chain for ATP production. Moreover, CoQ10 has strong antioxidant properties that may overcome cellular antioxidants (tocopherol and resveratrol) in its actions against ROS attacks (Tiwari et al. 2021).

CoQ10 was introduced into animal feeds for the treatment of various OS-induced organ dysfunctions, including cattle (Wafa and El-Nagar 2021), rabbits (Elokil et al. 2019), and birds (Sharideh et al. 2020), horses (Nemec Svete et al. 2021), rats (Delkhosh et al. 2021) and humans (Nadjarzadeh et al. 2014). CoQ10 supplementation enhances testicular function and fertility in aged breeding roosters and heat-stressed rabbits by lowering OS, increasing total antioxidant capacity and testosterone levels, and upregulating testicular melatonin receptors (Elokil et al.^2019^; Sharideh et al.^2020^).

Evaluation of TBF using colored-Doppler ultrasonography has become a useful, precise, and non-invasive tool for predicting testicular functionality via assessment of the testicular artery’s impedance against blood flow. In an oxidative state, the vascular endothelium becomes more vulnerable to ROS attack especially superoxide anion, which affects nitric oxide (NO, vascular tone regulator) synthesis with a subsequent decrease in testicular blood perfusion. Several studies (Gloria et al. 2020; Samir et al. 2020; Abdelnaby et al. 2021) have reported a strong relation between Doppler indices and testicular functions (steroidogenesis and spermatogenesis).

Based on the strong antioxidant capacity of CoQ10 and the advantages of free diffusion through the mitochondrial membrane and elimination of cradle-generated ROS; we hypothesized that summer oral CoQ10 supplementation could weaken OS induced by heat stress and improve reproductive performance in the bucks. To date, this is the first study to examine the effects of oral CoQ10 supplementation on testicular hemodynamics and semen quality in goat bucks under HS conditions.

## Materials and methods

### Animals and management

Ten Baladi goat bucks, with average body weight and age (49 ± 2.9 kg; 3.4 ± 0.6 y, respectively), were used in this study. These bucks were thoroughly examined before being used in the experimental procedures to exclude any buck with cardiovascular and andrological problems suck as orchitis, based on general examination of the vital signs and Doppler ultrasound scanning of the reproductive system. They were housed in a paddock throughout the experimental time points, with the availability of a 20 m^2^ shaded slatted area and exposed to natural daylight, ambient temperature, and relative humidity in the summer season in Egypt. The bucks were offered a balanced diet following the NRC instructions, composed of 400 g/ head/day of ready-to-eat pelleted concentrates (18% crude protein) and 1.25 kg/head/day of green roughage. The feed ingredients based on the manufacturer were yellow corn, wheat bran, cotton cake, sunflower cake, soybean meal, gluten feed of 16% and 22%, molasses, lime, NaCl, mineral salts, vitamins, and anti-mycotoxins; while, the feed chemical compositions were crude protein (18%), fat (2%), ash (9%), fiber (15%), and total digestible nutrients (65%). Fresh water and mineral licks were available for the bucks *ad libitum.* Periodical vaccination and deworming protocols were implied, following the instructions of the general authority of veterinary services in Egypt.

### Heat stress assessment

**Fig. 1:**
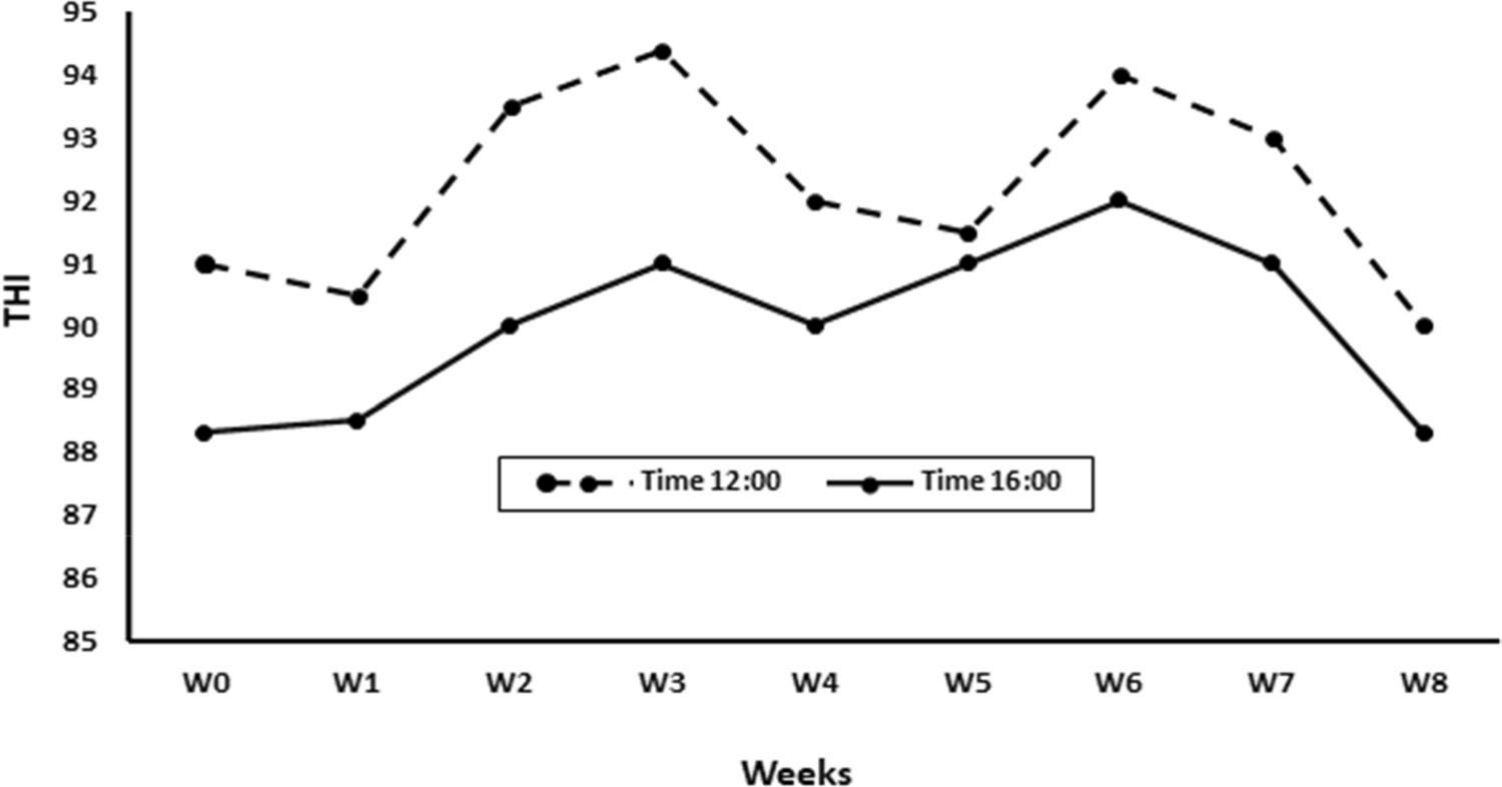
Temporal changes of the temperature humidity index (THI: 12:00 and 16:00) throughout the study timeline (W0-W8) in Giza city, Giza governorate, Egypt. The Calculated THI was based on the equation proposed by (Kendall and Webster 2009): THI = (1.8 × T + 32) − [(0.55 − 0.0055 × RH) × (1.8 × T − 26)]

Temperature humidity index (THI) was followed to assess whether the examined bucks were under heat stress conditions or not (Habeeb et al. 2018). THI was calculated based on temperature (T) and relative humidity (RH) records obtained from the Egyptian meteorological authority for the study period (June to August 2021), and territory (Giza city, Giza governorate, Egypt). The obtained T and RH data were for two times/day (12:00 and 16:00) throughout the study timeline (36–40 °C and 60–70%, respectively). The equation proposed according to a previous study (Kendall and Webster 2009) was used to calculate the THI i.e. THI = (1.8 × T + 32) − [(0.55 − 0.0055 × RH) × (1.8 × T − 26)]. THI results (Fig. 1) ranged from 88.3 to 94.8 throughout the study. Therefore, the bucks under examination have been considered under severe heat stress conditions (El-Tarabany et al. 2017).

### Experimental design

The present work has been carried out at the research farm of the Theriogenology Department, Faculty of Veterinary Medicine, Cairo University. This study was performed in the hottest months in Egypt (June to August). Bucks were divided into two equal groups; the first group (*n* = 5, Age: 3.6 ± 0.45 y; BW: 50.4 ± 2.2) received the programmed daily diet only (control group, CON), while the other group (*n* = 5, Age: 3.34 ± 0.35 y; BW: 48.4 ± 1.5) received the programmed daily diet plus oral administration of coenzyme Q10 at a dose of 3 mg/kg BW (CoQ10; Arab company for pharmaceutical and medicinal plants, Heliopolis, Egypt; Coenzyme Q10 Forte 100 mg/soft gelatin capsule) for six consecutive weeks (W1-W6). The CoQ10 dose was designed according to a previous study on goats (El-Ela et al. 2017). The supplemented and control bucks have been examined one week before (W-1) and on the first day of CoQ10 supplementation (W0) and once a week for eight consecutive weeks (W1-W8). The last two examinations (W7-W8) were to evaluate the post-treatment effect after the stoppage of CoQ10 supplementation. During the experimental time points (W-1-W8), the bucks have been examined for testicular hemodynamics [resistive index (RI) and pulsatility index (PI), endpoint of velocity (EDV), peak point of velocity (PSV), and colored area toward the testes/ pixels(CA)], testicular traits [testicular echotexture (TE) and testicular volume (TV)], reproductive hormones (FSH, LH, testosterone (T), estradiol 17β (E2)], nitric oxide (NO) and semen quality parameters [sperm progressive motility (SPM), viability (SV), normal morphology (NS) and sperm cell concentration (SCC)].

### Evaluation of testicular blood flow

**Fig. 2:**
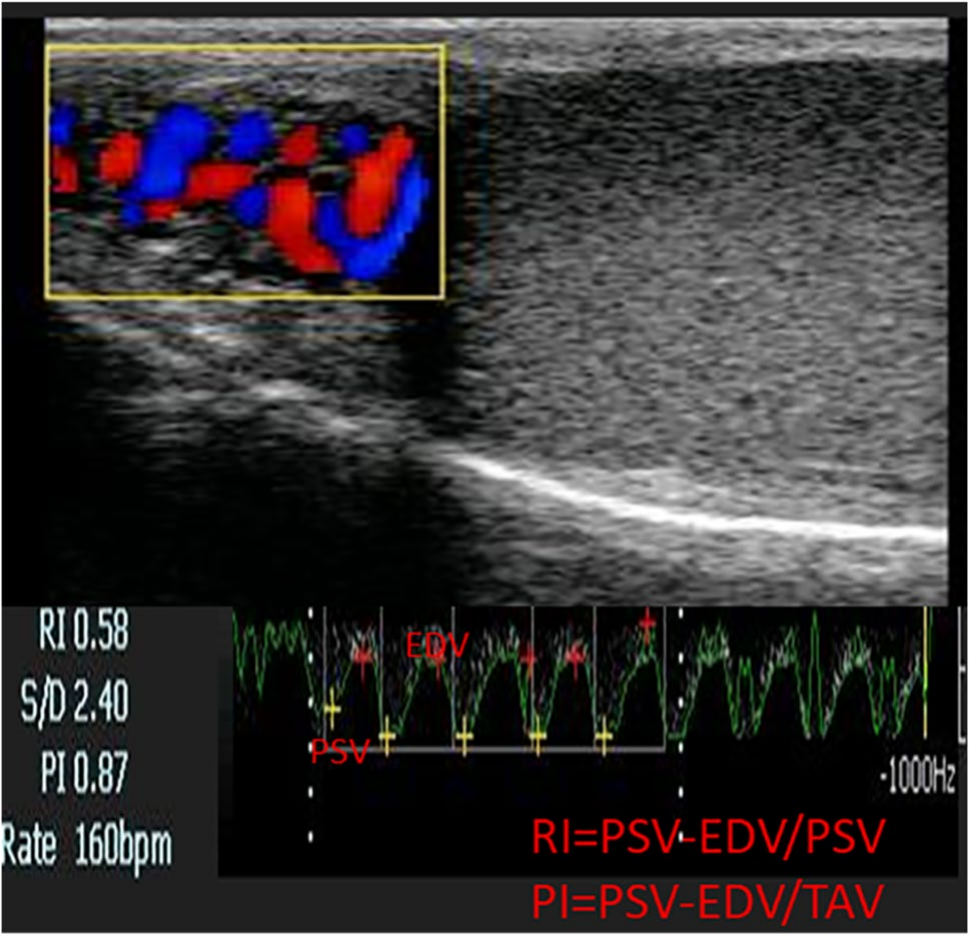
Color mode ultrasonograms of the pampiniform plexus showed colored areas. Note: Larger areas were colored blue to represent blood flow toward testes away from the probe, then a pulsed wave Doppler was activated to form the spectral graph. PSV = peak point of velocity, EDV = endpoint of velocity, TAV = time average velocity, RI = resistive index, and PI = pulsatility index

All the ultrasound scanning procedures throughout the studied time points were carried out by the same qualified operator. Doppler ultrasonography (EXAGO, Echo Control Medical [ECM], France), equipped with a 5–7.5 MHz linear array probe, was used for the assessment of the testicular hemodynamics (TH). Before the ultrasound examination, the animals were controlled without a tranquilizer to eliminate its impact on TH, and the scrotal hair on both sides of the testes and the spermatic cord was clean-shaven. Also, the Doppler sets including gate (0.5 mm), filter (50 Hz), and the angle between the Doppler beam and the longitudinal axis of the supra-testicular (STA) artery (< 60°) were adjusted and kept constant during the study (Samir et al. 2015). Doppler probe was placed vertically on a side of each testis and moved upward for recognition of the vascular network of the spermatic cord. For the STA insight (which appeared convoluted at the proximal pole of the testis) from the venous network, the waveforms (systole and diastole) of the STA clear the issue (Samir et al. 2020). Testicular hemodynamic parameters were estimated including resistive and pulsatile indices (RI and PI), the peak point of velocity (PSV; cm/s), and endpoint of velocity (EDV; cm/s; Fig. 2). The pampiniform vascularization was assessed by measuring the number of colored area/pixels (CA, pixels) either away (blue) or toward (red) the probe, the pre-saved colored Doppler image was operated using Adobe Photoshop cc × 64 software for determining the number of colored areas/pixels (EL-Sherbiny et al. 2022a). The testicular volume was calculated using the following ellipsoid equation = 4/3π abc; where length/2 (a) × width/2(b) × height/2(c) (Love et al. 1991). Echotexture was measured by the Adobe Photoshop CC program as previously described (Brito et al. 2012). Ultrasound measurements were repeated at least three times for results’ validity assurance.

### Assaying of circulating hormones and NO levels

Blood samples were collected, just before ultrasound scanning, from the jugular vein into heparinized vacutainer tubes (5 ml). All samples were centrifuged at 3000 rpm for 15 min, and then the harvested plasma was stored (− 20 ℃) until further assessment. Species-specific T, FSH, LH, and E2 were assayed from plasma samples by commercial ELISA (SunLong Biotech Co., LTD CHINA) kits with intra- and inter-assays variation coefficients were ≤ 10 and ≤ 12% for all measured hormones. For NO measuring, 100 µL of serum samples were mixed with equal volumes of Griess reagent [N-(l-naphthyl) ethylenediamine and sulphanilamide] and incubated for 18–20 min at room temperature. The optical density was recorded using a spectrophotometer (543 nm wavelength) and the NO (µM/L) concentration was calculated (Abdelnaby et al. 2021). Hormonal and biochemical analyses were repeated at least two times for valid results assurance.

### Semen quality assessment

On the day of ultrasound scanning and blood sampling, semen samples were collected using an artificial vagina (42 °C). The collected samples were transferred immediately to the laboratory and held in a water bath (35 °C) for further evaluation. Each semen sample was divided into two parts, the first portion for sperm microscopical evaluation and the second one for biochemical evaluation of TAC, CAT, ALT, and MDA. To exclude the negative effects of long-time sexual rest, the semen quality data regarding the week before CoQ10 supplementation (W-1) was discarded (Zarazaga et al. 2010).

### Microscopical evaluation

Sperm progressive motility (SPM, %), viability (SV, %) and normal morphology (NS, %) were examined subjectively using a heated-stage (38 °C) light microscope (Olympus, Optical co., Ltd., Tokyo, Japan), while, SCC (10^9^ sperm cell/ ml) was recorded using the improved Neubauer hemocytometer (Mahmoud et al. 1997).

For sperm progressive motility, a diluted [sodium citrate dihydrate 2.9% (1:20; v/v)] semen sample was loaded on a pre-warmed glass slide (37 °C) and cover-slipped. The spermatozoa that had forward progressive motility were recorded and expressed in percent. The sperm viability and normal morphology percentages were examined employing the eosin-nigrosin staining (Sprecher and Coe 1996) technique (5 g eosin, 30 g nigrosin, 8.7 g sodium citrate dihydrate dissolved in 300 ml distilled water in a boiling water bath for at least 15 min, and filtered to be homogenous and clear). A diluted (1:20; v/v) semen drop was thoroughly mixed with a pre-warmed (37 °C) stain drop (1:2 v/v) on a pre-warmed glass slide and smeared gently by another slide. The air-dried slide was examined using a light microscope (magnification: 400 ×). For sperm viability assessment (300 sperm were assessed/slide), spermatozoa with defective plasma membranes appeared pink in color; while those with integrated plasma membranes were not stained (colorless). For normal morphology assessment (300 sperm were assessed/slide) using an oil immersion lens (magnification: 1000 ×), spermatozoa that had abnormalities in heads (e.g. detached, pyriform, giant, dwarf, or tapered), midpiece (bowed), cytoplasmic droplet (proximal or distal), and tail (coiled, bent, or double) were considered abnormal. Duplicate smears were followed for all the studied semen parameters.

### Seminal plasma oxidative markers (TAC, CAT, and MDA) and ALT activity

For obtaining the seminal plasma (SP), semen samples were centrifuged (4 °C) at 2000 g for 15 min, followed by storage of the harvested SP at -20 °C till later measurement. To exclude the inter-assay variations, all the biochemical evaluations (W0-W8) were performed on the same day after the end of the study. Colormetrically using a spectrophotometer, commercial research kits were used for assaying the levels and activities of TAC (mM/L), CAT (U/L), MDA (mM/mL) (Biodiagnostics, Dokki, Giza, Egypt), and ALT (U/ml; Spectrum-diagnostic, Obour city, Cairo, Egypt) specifically at 510, 520, 534, and 505 nm wavelengths, respectively, following the manufacturer’s instructions (Fathi et al. 2021).

For MDA analysis, the principle of methodology depends on thiobarbituric acid (TBA) reacting with malondialdehyde in an acid medium for 30 min at 95 °C to produce a TBA product (pink product). The absorbance of the product was evaluated at 534 nm. The estimation was done according to the manufacturer’s instructions for the commercial kit following the principle described by Satoh (1978) and Ohkawa et al. (1979).

The estimation of TAC was achieved via the reaction of the sample’s antioxidants with a specified quantity of exogenously supplied hydrogen peroxide (H_2_O_2_). The antioxidants in the sample eliminate a certain amount of the supplied H_2_O_2_. The residuum H_2_O_2_ is estimated colorimetrically by an enzymatic reaction which involves the transformation of 3,5, dichloro –2– hydroxy benzenesulfonate to a colored product that is read against distilled water at 505 nm. TAC was estimated using commercial test kits following the principle described by Koracevic et al. (2001).

### Statistical analysis

Firstly, the raw data were checked for normality using the Shapiro–Wilk test. The differences between the right and left testis regarding the Doppler findings were non-significant; therefore, the data of both testes were pooled and the means in each time point were presented. The biological repetition was three times for ultrasound measurements, two times for semen quality, hormonal and biochemical assessments. The differences between control and CoQ10 groups (treatment effect), in terms of testicular hemodynamics values (RI, PI, EV, PV, and CA), circulating hormones (FSH, LH, T, and E2), NO, semen quality traits (SPM, SV, NS, SCC), seminal oxidative biomarkers (TAC, CAT, and MDA), and ALT activity throughout the studied time points (time effect) and combined treatment time effect were analyzed using repeated-measures ANOVA test, followed by Tukey *post hoc* test. Probability less than 5% was considered a significant result. The values were presented as means ± standard error of the mean (SEM). The Statistical Package for Social Sciences (SPSS®; version 20; Chicago, USA) was used for all statistical analyses.

## Results

### B- mode ultrasound evaluations

**Fig. 3:**
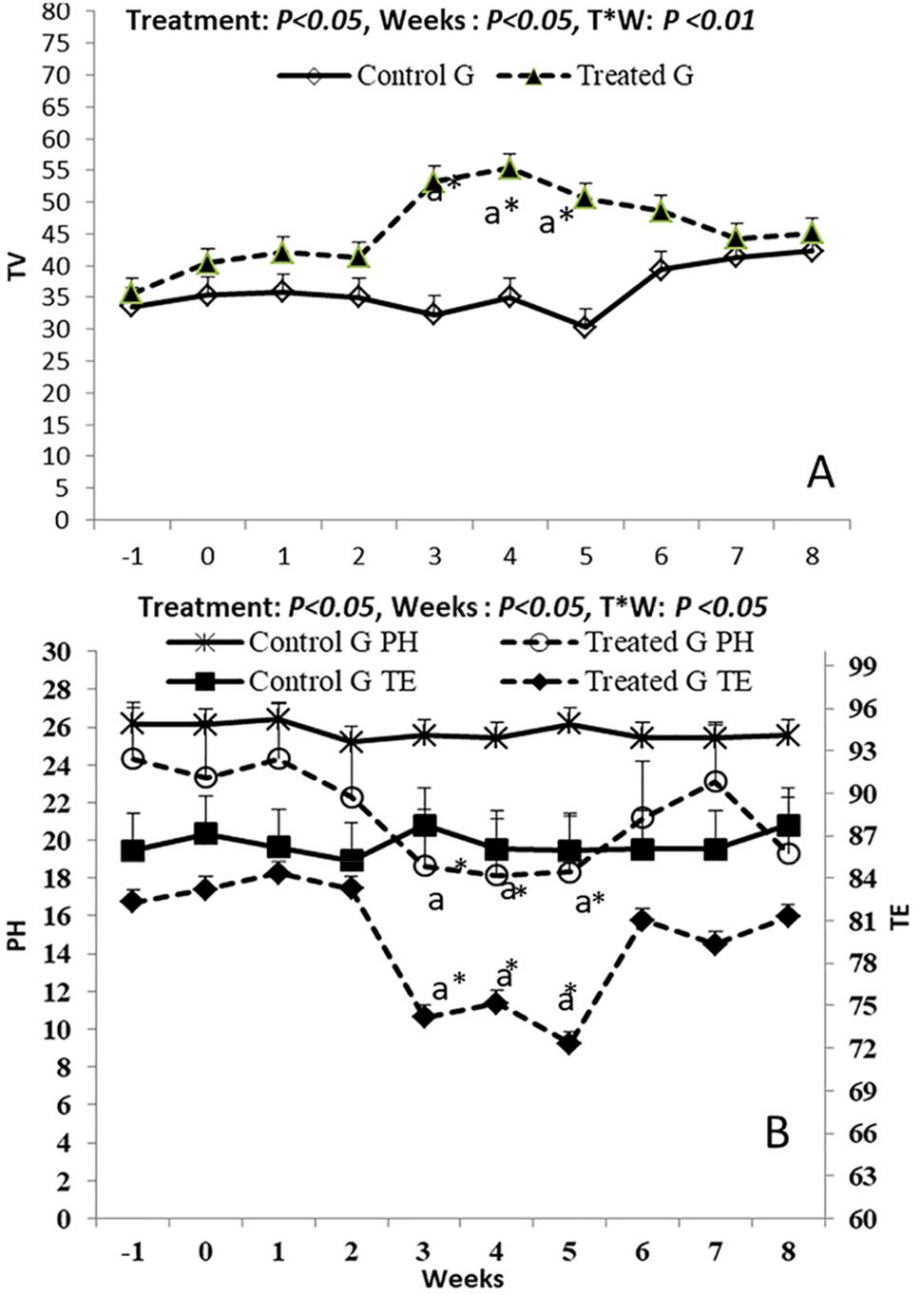
Alterations in testicular volume (cm^3^; A), and testicular echotexture (B) in form of pixel heterogeneity (PH, SdNPVs) and testicular echogenicity (TE, NPVs). ^a^ value is significantly different at *P* < 0.05 compared with the control and coenzyme Q10 males, while the * value is significantly different at *P* < 0.05 between the two groups at the same time point

The coenzyme Q10 supplement affected the TV dimensions. Testicular volume (cm^3^) elevated significantly (*P* < 0.05) from W 3 (53.24 ± 2.74) till W 5 (50.65 ± 2.55) as shown in (Fig. 3A), while the time and treatment*time interaction had shown a significant (*P* < 0.01) difference in the TV values, while both TE and pixel heterogeneity (PH) means were affected by the treatment by declination from W 3 (74.148 ± 1.22 for TE, and 18.66 ± 1.01for PH) till W 5 (72.21 ± 0.11 for TE, and 18.33 ± 0.77 for PH), while the time effect did not significantly affect both parameters compared to the CON group (Fig. 3B).

### Testicular hemodynamics evaluation

The CoQ10 supplementation affected the main testicular artery vascularity as shown in Table 1. Overall, the treatment effect (CoQ10 vs CON) showed significant (*P* < 0.01) differences in the values of RI, PI, PSV, and EDV; while the time effect had significant (*P* < 0.01) differences in RI, PI, PSV, and CA without affecting the EDV. However, the treatment*week interaction had a non-significant effect on all Doppler values. Both Doppler indices (RI and PI) decreased (*P* < 0.001) from W3 (0.62 ± 0.02 and 0.71 ± 0.02) till W 6 (0.49 ± 0.02 and 0.61 ± 0.02). Concurrently, both PSV and CA markedly increased (*P* < 0.001) from W3 (19.45 ± 0.05 and 1008.3 ± 6.32) till W6 (20.99 ± 0.07, and 1321.5 ± 6.35), respectively.

**Table 1:** Supra testicular artery Doppler parameters including PI, RI, PSV, EDV, and colored areas (CA) away probe in the pampiniform plexus in CoQ10 supplemented (CoQ10; *n* = 5) versus control (CON; *n* = 5) group at the studied time points (W0-W8) under heat stress conditions (THI: 88.3–94.4)

W	PI		RI		PSV cm/s		EDV cm/s		CA away probe toward testes (pixel)	
	CoQ10	CON	CoQ10	CON	CoQ10	CON	CoQ10	CON	CoQ10	CON
W -1	0.88 ± 0.01	0.92 ± 0.01	0.71 ± 0.01	0.72 ± 0.01	15.58 ± 0.01	15.24 ± 0.11	4.32 ± 0.04	4.33 ± 0.09	655.2 ± 22.31	593.2 ± 30.69
W0	0.91 ± 0.01	0.89 ± 0.01	0.74 ± 0.02	0.72 ± 0.01	15.98 ± 0.04	15.56 ± 0.05	4.51 ± 0.01	4.40 ± 0.07	645.2 ± 6.32	662.6 ± 19.63
W1	0.92 ± 0.02	0.90 ± 0.01	0.73 ± 0.02	0.71 ± 0.01	15.66 ± 0.04	15.56 ± 0.06	4.64 ± 0.02	4.41 ± 0.07	711.3 ± 6.33	633.4 ± 34.18
W2	0.90 ± 0.01	0.89 ± 0.01	0.71 ± 0.02	0.69 ± 0.01	15.64 ± 0.12	15.45 ± 0.12	4.88 ± 0.04	4.78 ± 0.02	645.2 ± 3.25	692.6 ± 22.83
W3	0.71 ± 0.02*	0.91 ± 0.01	0.62 ± 0.02*	0.72 ± 0.01	19.47 ± 0.05*	15.61 ± 0.05	4.45 ± 0.01	4.33 ± 0.05	1008.3 ± 6.32*	703.2 ± 23.83
W4	0.68 ± 0.02*	0.87 ± 0.01	0.59 ± 0.02*	0.71 ± 0.01	19.58 ± 0.07*	15.48 ± 0.07	4.64 ± 0.03	4.40 ± 0.03	1142.2 ± 2.36*	848.4 ± 35.17
W5	0.64 ± 0.02*	0.87 ± 0.01	0.54 ± 0.02*	0.71 ± 0.01	20.15 ± 0.05*	15.48 ± 0.06	4.68 ± 0.01	4.38 ± 0.05	1258.4 ± 5.32*	855.6 ± 9.37
W6	0.61 ± 0.02*	0.89 ± 0.01	0.49 ± 0.02*	0.72 ± 0.01	20.99 ± 0.07*	15.54 ± 0.07	4.64 ± 0.01	4.35 ± 0.03	1321.5 ± 6.35*	882.2 ± 21.01
W7	0.88 ± 0.01	0.87 ± 0.02	0.69 ± 0.01	0.72 ± 0.01	15.21 ± 0.06	15.57 ± 0.06	4.47 ± 0.02	4.40 ± 0.04	875.2 ± 15.21	886.2 ± 22.43
W8	0.91 ± 0.02	0.89 ± 0.01	0.70 ± 0.01	0.71 ± 0.01	15.01 ± 0.07	15.55 ± 0.07	4.66 ± 0.02	4.36 ± 0.08	888.2 ± 11.32	748.2 ± 12.31

*PI* pulsatility index, *RI* resistive index, *PSV* peak point of velocity, *EDV* end point of velocity, *W* weeks, and *CA* colored areas. Data presented as mean ± SEM. *Values in each measure are different at least at *P* < 0.05 between the two groups

### Hormonal (T, E2, FSH, and LH), and NO assay

**Fig. 4:**
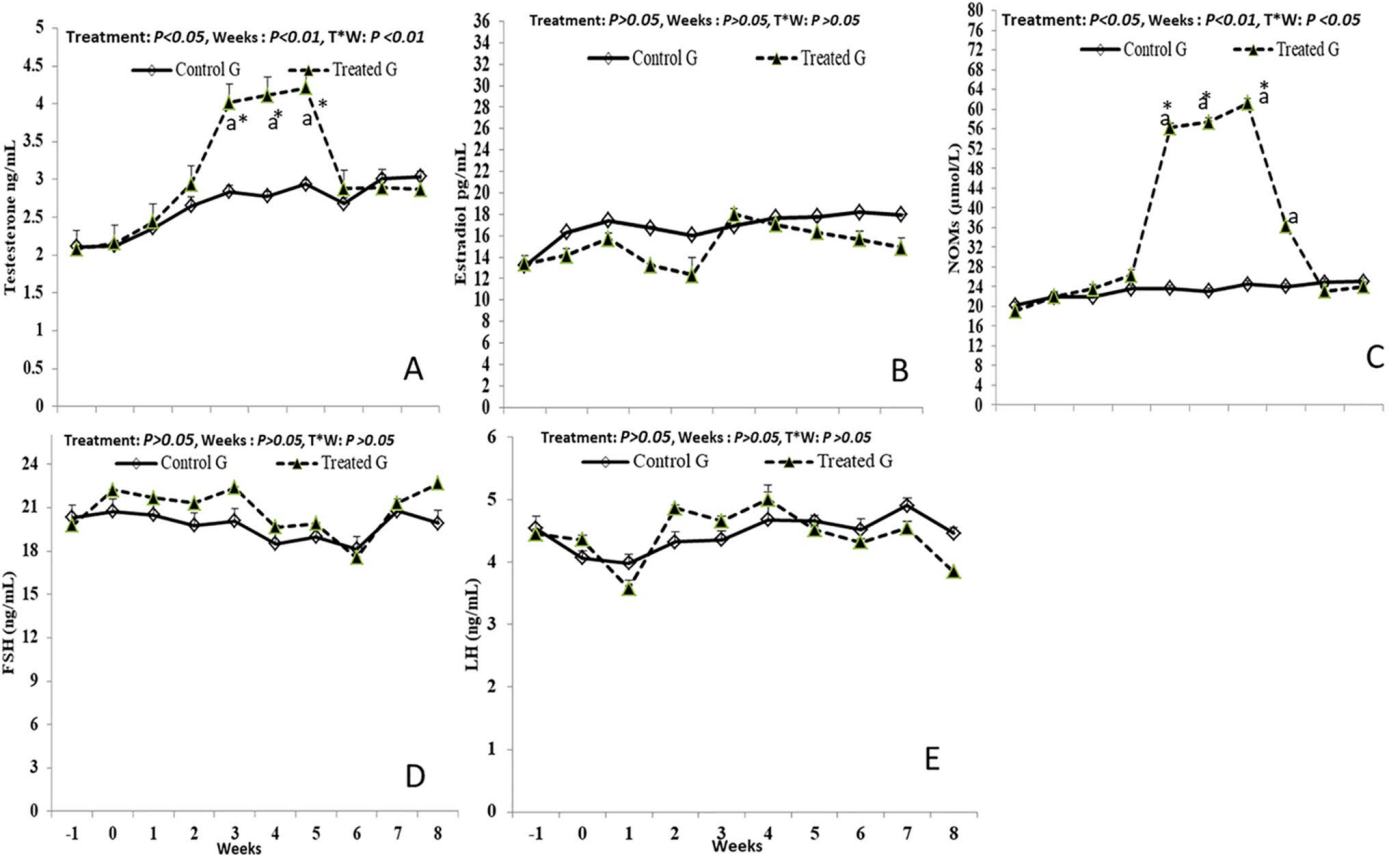
Alterations in plasma levels of testosterone (T; ng/mL; A), estradiol (E2; pg/mL; B), serum nitric oxide levels (NOMs, µmol/L; C), follicle-stimulating (FSH; ng/mL; D), and luteinizing hormones (LH; ng/mL; E) in male bucks that received coenzyme Q10 compared to control group. Values are presented as means ± SEM. ^a^ value is significantly different at *P* < 0.05 compared with the control and coenzyme Q10 males, while the * value is significantly different at *P* < 0.05 between the two groups at the same time point

The CoQ10 treatment and time*treatment interaction effects (CoQ10 vs CON) had a non-significant difference in the plasma levels of E2, FSH, and LH (Fig. 4B, D & E). Moreover, The levels of T and NO increased significantly (*P* < 0.01) in the CoQ10 group compared to the CON group (Fig. 4A & C). The T (ng/mL) and NO (µM/L) values increased significantly with time (*P* < 0.01) from W3 (4.02 ± 0.14 and 56.25 ± 0.73) till W5 (4.21 ± 0.02 and 61.21 ± 0.08), respectively. Furthermore, the time*treatment interaction had significant differences in T (*P* < 0.01) and NO (*P* < 0.05) levels.

### Semen quality parameters

The data showing the effect of supplemental dietary CoQ10 on the semen characteristics of heat-stressed goat bucks (CoQ10 vs CON; W0-W8) is presented in Table 2. The CoQ10 supplementation improved significantly (*P* < 0.01) the percentages of SPM, SV, and NS starting at W3 till W6. There was a carrying-over effect of treatment on SCC till W8 compared to the CON group.

**Table 2:** Semen characteristics (Progressive motility, viability, normal morphology, sperm cell concentration) in CoQ10 supplemented (CoQ10; *n* = 5) versus control (CON; *n* = 5) group at the studied time points (W0-W8) under heat stress conditions (THI: 88.3–94.4)

W	Progressive motility %		Viability %		Normal morphology %		Sperm cell concentration 10^9^/mL	
	CoQ10	CON	CoQ10	CON	CoQ10	CON	CoQ10	CON
W0	72.20 ± 1.33	70.00 ± 1.84	82.40 ± 1.11	83.20 ± 1.45	83.60 ± 2.66	82.60 ± 1.88	2.22 ± 0.02	2.26 ± 0.55
W1	69.00 ± 1.77	69.00 ± 1.55	76.00 ± 1.44	79.80 ± 1.65	82.40 ± 0.87	82.40 ± 0.88	2.45 ± 0.01	2.33 ± 0.07
W2	73.00 ± 1.65	70.00 ± 1.75	81.00 ± 0.54	79.60 ± 2.33	83.00 ± 0.58	84.40 ± 0.94	2.21 ± 0.01	2.24 ± 0.05
W3	78.00 ± 1.33*	73.00 ± 2.04	88.60 ± 1.27*	81.00 ± 1.45	89.40 ± 0.84*	83.60 ± 0.85	2.35 ± 0.01	2.26 ± 0.07
W4	79.00 ± 1.18*	71.00 ± 1.58	90.40 ± 1.22*	82.60 ± 1.05	88.40 ± 0.54*	83.40 ± 0.88	2.29 ± 0.02	2.14 ± 0.04
W5	80.00 ± 2.14*	68.00 ± 2.04	91.60 ± 1.44*	81.60 ± 1.97	89.60 ± 0.44*	83.00 ± 0.77	2.11 ± 0.01	1.97 ± 0.04
W6	81.00 ± 1.11*	72.00 ± 1.52	91.60 ± 1.41*	80.80 ± 1.55	91.40 ± 0.54*	82.60 ± 0.68	2.45 ± 0.01*	1.97 ± 0.05
W7	68.20 ± 1.44	69.20 ± 1.45	81.20 ± 1.21	79.80 ± 1.71	81.00 ± 0.74	83.00 ± 0.55	2.52 ± 0.02*	2.03 ± 0.04
W8	73.60 ± 1.01	72.40 ± 1.12	78.60 ± 1.74	80.80 ± 1.57	84.40 ± 0.55	84.00 ± 0.28	2.61 ± 0.01*	2.15 ± 0.05

*THI =* temperature humidity index, *W =* weeks. Data obtained as mean ± SEM. *Values in each measure are different at least at *P* < 0.05 between the two groups. The treatment effect (CoQ10 versus CON) showed a significant difference in sperm progressive motility % (*P* < 0.001), viability (*P* < 0.0001), and concentration (*P* < 0.0001), while the time effect showed a significant (*P* < 0.01) difference in sperm progressive motility, viability, normal morphology, and concentration

### Seminal plasma oxidative markers(MDA, TAC, and CAT) and ALT activity

The seminal plasma levels of MDA and TAC, and activities of CAT and ALT enzymes (CoQ10 vs CON) studied at different time points (W0-W8) under heat stress conditions are presented in Table 3. There were significant (*P* < 0.01) effects of treatment (CoQ10 vs CON) and time on the values of MDA and TAC, and activities of CAT and ALT. There were marked increases in the levels of TAC (W2-W6) and CAT (W4-W8) activity, and decreases in the values of MDA (W5-W8) and activity of ALT (W4-W7) in the CoQ10 group compared to the CON group.

**Table 3:** Seminal plasma levels of malondialdehyde (MDA) and total antioxidant capacity (TAC) and activities of catalase (CAT) and alanine aminotransferase (ALT) enzymes in CoQ10 supplemented (CoQ10; *n* = 5) versus control (CON; *n* = 5) group at the studied time points (W0-W8) under heat stress conditions (THI: 88.3–94.4)

W	MDA (nM/mL)		TAC (mM/L)		CAT (U/L)		ALT (U/mL)	
	COQ10	CON	COQ10	CON	COQ10	CON	COQ10	CON
W0	2.76 ± 0.01	2.72 ± 0.03	0.63 ± 0.01	0.60 ± 0.04	266.49 ± 24.45	267.91 ± 23.72	41.13 ± 1.33	43.06 ± 0.51
W1	2.73 ± 0.05	2.78 ± 0.11	0.69 ± 0.01	0.63 ± 0.05	278.74 ± 28.32	280.58 ± 24.27	45.82 ± 2.41	44.34 ± 1.24
W2	2.62 ± 0.01	2.73 ± 0.01	0.84 ± 0.01^*^	0.64 ± 0.01	247.33 ± 15.97	256.01 ± 24.25	47.68 ± 2.65	43.62 ± 1.54
W3	2.69 ± 0.01	2.77 ± 0.02	1.27 ± 0.01^*^	0.64 ± 0.07	284.38 ± 26.58	296.42 ± 30.74	48.74 ± 2.01	43.52 ± 2.63
W4	2.52 ± 0.02	2.71 ± 0.01	1.29 ± 0.01^*^	0.88 ± 0.07	396.87 ± 24.31^*^	299.73 ± 46.13	36.24 ± 0.97^*^	43.36 ± 1.76
W5	2.41 ± 0.04^*^	2.77 ± 0.03	1.33 ± 0.01*	0.92 ± 0.06	446.84 ± 15.88^*^	284.40 ± 36.69	38.90 ± 1.74^*^	47.36 ± 0.84
W6	2.11 ± 0.05^*^	2.66 ± 0.04	1.35 ± 0.02*	0.96 ± 0.12	494.71 ± 24.65^*^	309.87 ± 33.29	34.48 ± 4.02^*^	49.78 ± 1.22
W7	2.04 ± 0.04^*^	2.68 ± 0.01	1.01 ± 0.02	0.94 ± 0.08	487.27 ± 15.62^*^	284.01 ± 33.57	34.64 ± 1.22^*^	44.16 ± 0.21
W8	2.02 ± 0.04^*^	2.65 ± 0.06	0.79 ± 0.01	0.84 ± 0.12	529.364 ± 22.31^*^	299.29 ± 31.80	46.76 ± 2.31	43.55 ± 0.22

*THI* temperature humidity index, *W* weeks. Data obtained as mean ± SEM.*Values in each measure are different at least at *P* < 0.05 between the two groups. The treatment (COQ10 vs CON) and time effects showed significant (*P* < 0.01) differences in MDA, TAC, CAT, and ALT

## Discussion

Environmental heat stress deteriorates animal productivity and fertility potential (El-Sherbiny et al. 2022b). Since oxidative stress is the main pathway through which HS exerts its destructive impacts on male reproductive functions, numerous studies have reported that dietary supplementation of antioxidants could ameliorate HS-induced male infertility (Elokil et al. 2019; Sharideh et al. 2020; El-Sherbiny et al. 2022c; Fadl et al. 2022c). Mitochondria is the site where free radicals are extensively generated and induce an OS cascade. Therefore, mitochondrial-targeted antioxidants such as CoQ10 could be more beneficial than cytosolic antioxidants.

In the present study, there were marked changes in the STA Doppler indices of the CoQ10 supplemented males compared to the control group. These changes could be interpreted by a decrease in vascular resistance and higher testicular blood perfusion (Gill 1985; Abdelnaby et al. 2022; Hashem et al. 2022). It is well-known that heat-stressed males suffer lower testicular blood flow and functions (Hedia et al. 2020). The hypoxic status of the heat-stressed testicular tissues might be improved by increasing TBF. In the current study, the mechanisms by which CoQ10 nutritional supplements could increase TBF were not specified; however, different perspectives may explain these changes. Firstly, CoQ10 is an indispensable regulator of mitochondrial functions especially the oxidative phosphorylation process and ultimately ATP production (Sandhir et al. 2014). Secondly, the alleviation of heat-stress-induced endothelial dysfunction might be the first explanation for increased TBF in our study and CoQ10 could be accumulated in the vascular endothelium, following an intravenous administration, and induced NO-dependent aortic vasodilatation in rats (Kozaeva et al. 2017). An earlier study also reported that dietary CoQ10 supplements ameliorated diabetes mellitus-induced endothelial dysfunction of the brachial artery in humans (Watts et al. 2002). Improving the endothelial functions in oxidative stress conditions could be explained via its antioxidant capability which indirectly increases NO bioavailability through inactivation of superoxide anion radicals and inhibition of peroxynitrite formation (highly pro-oxidant compound generated via NO and superoxide anion combination). Indeed, CoQ10 administration alleviates the deteriorative impacts of ischemia/reperfusion injury in rats (Arda et al. 2021). The elevated NO levels noted in the present study inferred the protective role of CoQ10 in endothelial functions and synthesis of NO and ultimately higher testicular perfusion.

Electronic measurement of testicular size and its volume has been a reliable tool for male fertility assessment because it provides actual testicular parenchyma morphometric measurements without the need for additional scrotal and epididymal dimensions. In this study, there was a noticeable increase in the testicular volume. This improvement is likely due to the higher blood perfusion that might affect the fluidity of the testicular tissues (EL-Sherbiny et al. 2022d). CoQ10 has a protective effect on the seminiferous tubules' diameter and epithelial height and thickness (Sharideh et al. 2020). A recent study reported the treatment of heat-stressed rats with CoQ10 reversed all the detrimental impacts of heat stress on testicular tissues including irregularity of seminiferous tubules and damaged spermatogonia, spermatocytes, and spermatids as well as decreased testicular weight via its anti-apoptotic and anti-oxidative properties (Delkhosh et al. 2021).

Assessment of echogenicity of the testicular parenchyma provides a realistic information regarding the testicular histomorphology and fluidity (Camela et al. 2019). In this study, there were significant decreases in testicular echotexture (TE) (W3-W5), in the CoQ10 group, followed by gradual increases onward to be non-significant compared to the control group. Interestingly, the echogenic changes of testicular parenchyma were concurrent with the changes noted in the blood flow and cellular part of the testicular tissues. It has been reported that TE is inversely correlated with the blood flow and positively with the cellular matrix of the testes (Giffin et al. 2009). Therefore, higher TBF explains the lower TE values, whereas, the higher sperm cell concentration in the last three weeks of the experiment may interpret the elevated TE values. These outcomes support the concept that TE is affected by testicular dynamics either in blood flow or spermatogenesis and is useful as an indicator in the breeding soundness evaluation of male animals (El-Sherbiny et al. 2022e).

Monitoring the circulating reproductive hormones offers a deep insight into the physiological functions of the testes as well as the pituitary–gonadal axis (Samir et al. 2020; Mandour et al. 2022). In the current study, there were marked increases in the T values of the CoQ10 treated compared to untreated males. This improvement might be linked to CoQ10 properties a bioenergetics compound as it has a pivotal role in the electron transport chain and energy production in mitochondrial membranes of the testicular Leydig cells, which affects its activity and productivity (Delkhosh et al. 2021). Secondly, CoQ10 could protect testosterone-producing cells from oxidative stress via ROS scavenging and membrane lipid peroxidation inhibition (Elokil et al. 2019). Moreover, the CoQ10 can diminish the heat-stress-induced cellular apoptosis which is evident by Bcl2 (anti-apoptotic biomarker) up-regulation and BAX and caspase3 (apoptotic biomarkers) down-regulation (El-Khadragy et al. 2020). Numerous studies support the outcomes of the present study under testicular stress conditions in rats such as lead acetate and lipopolysaccharide toxicity (Song et al. 2017; El-Khadragy et al. 2020), chronic kidney disease (Tsao et al. 2021), and heat stress (Elokil et al. 2019) in rabbits. Unaltered levels of FSH, LH, and E2 during the experimental time points between CoQ10 supplemented males versus control need to be interpreted cautiously taking into consideration its pulsatile secretions. Contrarily, there was evidence that CoQ10 treatment decreased FSH and LH levels concurrent with elevated prolactin levels and these changes were attributed due to the anti-gonadotropic effects of higher prolactin levels (Nadjarzadeh et al. 2014; Alahmar et al. 2021). This discrepancy may be due to the species and conditions variations. Moreover, estradiol 17β is primarily synthesized in the Sertoli cells in the FSH-dependent pathway through testosterone conversion via aromatase activity (Dorrington et al. 1978). The non-significant changes in E2 levels are possibly due to the absence of a direct effect of CoQ10 on E2 synthesis (Sandhir et al. 2014).

Assessment of the seminal plasma oxidative biomarkers provides a clear insight through the sperm membrane integrities (Fadl et al. 2022a). In this study, there were marked increases in seminal plasma CAT activity and TAC, and a decrease in MDA concentration. These results suggest that CoQ10 supplementation improved seminal antioxidant power with subsequent higher membrane integrity evidenced by lower lipid peroxidation profile which is consistent with a previous study (Nadjarzadeh et al. 2014) and a study conducted in human medicine (Alahmar et al. 2021), roosters (Sharideh et al. 2020), and rats (Delkhosh et al. 2021). In addition, our results appear to indicate that CoQ10 rewarded HS-mediated depletion of seminal antioxidant capacity. The assaying of ALT activity considers a useful biomarker for sperm plasma membrane intactness because spermatozoa with damaged membranes release ALT in seminal plasma (Taha et al. 2000; Fadl et al. 2022b). There was a significant decrease in the seminal ALT activity in the present study indicating the higher integrity of the sperm plasma membrane following CoQ10 oral supplementation.

In the present study, there were remarkable improvements in semen quality traits at W3-W6 for sperm progressive motility, membrane integrity, and normal morphology, and at W6-W8 for SCC in the CoQ10 group versus the control group. Since subjective motility evaluation herein might have a wide error range among the examined animals and time points; a further study is recommended using advanced semen quality assessment techniques. Plenty of reports on roosters (Sharideh et al. 2020), rabbits (Elokil et al. 2019), men (Safarinejad 2009; Alahmar et al. 2021), and ducks (Fouda et al. 2021) favor the results of the present study. CoQ10 has a powerful antioxidant capacity and can regenerate endogenous antioxidant systems such as superoxide dismutase enzyme (Navas et al. 2007). Moreover, compared to fertile men, the seminal plasma of infertile men has lower levels of CoQ10 which could be increased via dietary supplementation and improved semen density and quality (Ghanbarzadeh et al. 2014; Nadjarzadeh et al. 2014). It has been reported that heat stress depletes the seminal plasma antioxidant capacity to combat the excess generation of free radicals (Elokil et al. 2019). The elevated TAC and CAT activities and decreased MDA and ALT concentrations noted in the present study inferred the role of CoQ10 in amelioration of heat stress in the perspective of semen quality via enhancement of seminal plasma antioxidant defense systems and lowering sperm membrane lipid peroxidation. Interpretation of the semen quality improvement in the present study, especially before the end of the spermatogenesis (47 days) is complex. However, some explanations may reveal the issue; firstly, daily sperm production in bucks is 20–30 × 10^6^ spermatozoa/g testicular tissue (the highest among domestic species) (Leal et al. 2004; Junior et al. 2011). secondly, average testicular and epididymal (head, body, and tail) sperm reserves were about 44, 8.8, 5.0, and 45.6 × 10^9^ (Daudu 1984). Thirdly, about 15–30% of the expected produced spermatids during the spermatogenesis process are lost especially at the two meiotic stages (Bilaspuri and Guraya 1986; Leal et al. 2004). Therefore, a massive number of spermatozoa already exist in the genital reserve rather than the newly produced ones. Moreover, the sperm maturation process alongside the HS conditions (Shahat et al. 2020) exacerbates the ROS generation which induces more sperm damage during the epididymal journey (Aitken and De Iuliis 2010). A recent study reported that dietary micronutrient administration for 12 days restores sperm OS and enhances semen quality in obese male mice (spermatogenesis ~ 34 days) (McPherson et al. 2019). Based on the above-mentioned evidence, oral coenzyme Q10 supplementation could alleviate the adverse effect of heat stress-mediated oxidative stress and enhance the semen quality (motility, viability, and morphology) in a period less than the whole length of the spermatogenesis process. In the present study, it was noted that the semen quality decreased after the end of CoQ10 supplementation (W7-W8); this decline may be attributed to the HS conditions that affected the sperm quality faster than the cold conditions.

## Conclusion

Oral Supplementation of CoQ10 during the summer season ameliorates the adverse effects of heat stress on the testicular hemodynamics and volume, testosterone production and sperm quality, and seminal antioxidant capacity which could improve goat breeding performance. Further studies are needed to investigate the role of CoQ10 supplementation in different male reproductive performance aspects such as sexual activity, more advanced semen quality techniques, and fertility potential.

## Data Availability

The data are available by the corresponding author upon a reasonable query.

## Declarations

### Concent to participate

All authors approved the final version of the manuscript.
